# Air Pollution and Pituitary Adenoma Pathogenesis: Unraveling Environmental Impacts on Neuroendocrine Function and Tumorigenesis

**DOI:** 10.3390/jox15030071

**Published:** 2025-05-12

**Authors:** Andre E. Boyke, Simon A. Menaker, Alberto Nunez, Keith L. Black, Vladimir A. Ljubimov

**Affiliations:** 1 Department of Neurosurgery, Cedars-Sinai Medical Center, 127 S San Vicente Blvd, Los Angeles, CA 90048, USAvladimir.ljubimov@cshs.org (V.A.L.); 2 Division of Otolaryngology-Head and Neck Surgery, Department of Surgery, Cedars-Sinai Medical Center, Los Angeles, CA 90048, USA

**Keywords:** pituitary adenoma, air pollution, neuroinflammation, hypothalamic–pituitary–adrenal axis, endocrine disruption, public health

## Abstract

Pituitary adenomas, although predominantly benign, can lead to significant clinical complications due to endocrine imbalances and mass effects on adjacent structures. Traditional research has focused on intrinsic factors like genetic mutations and hormonal dysregulation; however, emerging evidence implicates environmental pollutants—particularly urban air contaminants—in pituitary tumorigenesis. This review consolidates current findings on how chronic exposure to pollutants such as benzene, di(2-ethylhexyl) phthalate (DEHP), and polychlorinated biphenyls (PCBs) may trigger neuroinflammation, disrupt the hypothalamic–pituitary–adrenal (HPA) axis, and alter pituitary cell proliferation and hormone secretion. We explore mechanistic pathways involving inflammatory cytokines, oxidative stress, and microenvironmental modifications that contribute to neoplastic transformation and tumor progression. Epidemiological studies, supported by in vitro experiments, suggest that air pollutants not only initiate the development of pituitary adenomas but may also enhance the secretory activity of functioning tumors, potentially increasing their aggressiveness. Given the escalating global burden of air pollution and its far-reaching public health implications, further investigation is essential to elucidate these complex interactions. Advancing our understanding in this area could inform preventive strategies and therapeutic interventions aimed at mitigating the environmental impact on pituitary tumor behavior.

## 1. Introduction

The World Health Organization (WHO) estimates that ambient and indoor air pollution contributes to over 8 million deaths each year, combined. As mentioned, air pollution has been associated with diseases of the central nervous system (CNS), as well as neuroinflammation and abnormal neuropathological processes [1]. Research in both humans and animals demonstrates that neuroinflammation arises in response to various inhaled pollutants [2]. Growing evidence implicates air pollution as a chronic environmental factor in neuroinflammation, reactive oxygen species (ROS) production, and neurological injury, all of which can contribute to CNS pathology [3,4,5].

Direct evidence regarding primary brain tumors as a result of exposure to specific chemical agents is limited. This is in contrast to agents associated with tumors of the lung, colon, stomach, breast, uterus, and liver [6,7]. Furthermore, studies related to CNS tumors tend to largely deal with occupational risks rather than being based on investigating exposure to specific agents. Occupation in the electrical and electronic fields, oil refining, rubber, airplane manufacture, machining, farming, and pharmaceutical and chemical industries has been associated with increased CNS tumor risk [8]. Some culpable agents included benzene and other organic solvents, lubricating oils, acrylonitrile, vinyl chloride, formaldehyde, polycyclic aromatic hydrocarbons, phenol and phenolic compounds, and both ionizing and non-ionizing radiation [9].

Several studies were conducted on short- and long-term air pollutant exposure in rat and mouse brains. The brains of the experimental animals showed an overexpression of neuroinflammatory and/or tumor markers related to the development of primary brain tumors or neurogenerative conditions such as Alzheimer’s disease [9,10]. However, the knowledge and studies on pituitary tumors’ environmental dependence are still very limited.

Pituitary adenomas are predominantly benign pathological entities arising from the anterior pituitary gland and account for approximately 10–15% of primary brain tumors. They are generally classified as either functioning or non-functioning tumors, depending on the presence or absence of pathological hormone secretion [11]. Despite their benign nature, they may be associated with significant clinical sequelae, including hormonal dysregulation, visual deficits, pituitary apoplexy, and other symptoms related to mass effect on adjacent structures or invasive growth. While substantial progress has been made in understanding the endocrine mechanisms, genetic mutations, and clinical progression of these tumors, there is growing interest in the role of environmental factors in tumorigenesis. Among these, air pollution has emerged as a potential key contributor [12].

Air pollution is a complex mixture of harmful substances that includes particulate matter (PM_2.5_ and PM_10_), volatile organic compounds (VOCs), nitrogen oxides (NO_x_), sulfur dioxide (SO_2_), ozone (O_3_), and carbon monoxide (CO) [13]. The major sources of these pollutants include vehicle emissions, industrial manufacturing processes, fossil fuel combustion, agricultural activities, and residential heating. Fine particulate matter (PM2.5), in particular, can penetrate deep into the respiratory tract and enter the bloodstream, contributing to systemic inflammation and oxidative stress. VOCs and NO_x_ are prominently generated by traffic emissions and industrial solvents, while ozone is a secondary pollutant formed through photochemical reactions involving sunlight and precursor gases. Although persistent organic pollutants such as DEHP and PCBs are not classified as primary airborne pollutants, their inclusion in this review highlights the mechanistic pathways of endocrine disruption relevant to pollutant exposure more broadly. These insights complement the discussion on classic air pollutants implicated in systemic and neuroendocrine dysfunction.

In urban environments, the adverse effects of air pollution on cardiovascular and respiratory health are well documented. However, recent evidence also implicates air pollution in neuroendocrine dysfunction and potential tumorigenesis within the central nervous system [12]. The hypothalamic–pituitary axis, due to its critical regulatory functions and proximity to highly vascularized brain regions, may be particularly susceptible to the harmful effects of these pollutants [14].

The objective of this review is to provide an overview of the potential relationship between air pollution and pituitary adenoma pathogenesis, acknowledging the limited direct evidence in this emerging field. We aim to summarize existing studies, propose potential mechanisms, identify knowledge gaps, and integrate evidence from in vitro, in vivo, and epidemiological research. This review emphasizes the need to explore mechanisms linking environmental exposures to tumor development, addressing a critical gap in the current understanding. Articles were selected based on their relevance to the proposed mechanistic links between environmental pollutant exposure and pituitary tumorigenesis. Priority was given to peer-reviewed original research articles, in vitro and in vivo experimental studies, and systematic reviews published between 2000 and 2024, although seminal studies outside this range were included to provide historical context. The search strategy emphasized studies exploring both epidemiological associations and biological mechanisms, including inflammatory signaling pathways, endocrine disruption, and hypothalamic–pituitary axis dysregulation. Reference lists of key articles were manually screened to ensure comprehensive coverage of relevant findings.

## 2. Hypothalamic–Pituitary–Adrenal (HPA) Axis and Pituitary Pathophysiology

The normal physiological function of the hypothalamic–pituitary–adrenal (HPA) axis is the integration of signals from the central nervous system and peripheral organs to maintain endocrine homeostasis [15]. Hormones released from the hypothalamus, including corticotropin-releasing hormone, thyrotropin-releasing hormone, and gonadotropin-releasing hormone, play a key role in this process. These hormones travel through the hypothalamic–hypophyseal portal system to act upon cells within the anterior pituitary gland, a highly vascular structure that produces several key hormones: adrenocorticotropic hormone (ACTH), thyroid-stimulating hormone (TSH), luteinizing hormone (LH), follicle-stimulating hormone (FSH), growth hormone (GH), and prolactin. Each of these hormones significantly contributes to feedback loops with peripheral endocrine organs, regulating metabolism, the stress response, and other vital physiological processes [15]. In addition, the neurohormones oxytocin and antidiuretic hormone (ADH) are synthesized in the hypothalamus and stored in the posterior pituitary gland prior to secretion into the systemic circulation. Given the complex interplay between the hypothalamus and pituitary, disruption via inflammation or cellular proliferation may lead to endocrine abnormalities [16].

Regarding functioning tumors, prolactinomas are the most common subtype and cause hyperprolactinemia, resulting in symptoms like galactorrhea, menstrual irregularities, and infertility, among others. Somatotroph adenomas secrete growth hormone, leading to acromegaly in adults or gigantism in children. Corticotroph adenomas produce excessive ACTH, causing Cushing’s disease. Less common functioning adenomas include thyrotroph adenomas (excess TSH) and gonadotroph adenomas (excess LH/FSH). Non-functioning adenomas typically present with symptoms related to mass effect or invasive behavior. Compression of the optic chiasm may result in visual field deficits, while extension into adjacent structures, including the cavernous sinus or sphenoid bone, can contribute to headache or other neurological symptoms [11].

When considering the development, progression, and clinical behavior of pituitary adenomas, the surrounding tumor microenvironment (TME) has been found to play a critical role [17]. The TME is composed of communities of stromal cells, immune cells, and vascular networks which interact directly and indirectly with each other and with adjacent tumor cells. Immune cells in particular are known to be involved in pituitary adenoma invasiveness. For example, tumor-associated macrophages are pivotal in secreting cytokines such as interleukin-6 (IL-6) and vascular endothelial growth factor (VEGF), which act to promote proliferation and angiogenesis [18]. Elevated MMP (matrix metalloproteinase) activity facilitates extra-cellular matrix degradation, while cytokines promote proliferation and therefore may enable tumor invasiveness. Another key feature enabling these processes is hypoxia. As pituitary tumors grow, their expansion often surpasses their vascular supply, resulting in hypoxic regions. This upregulates hypoxia-inducible factor 1-alpha (HIF-1α), promoting angiogenesis and metabolic reprogramming to sustain tumor survival and proliferation [19]. Concurrently, oxidative stress within the hypoxic TME induces genomic instability, further driving tumor progression. These microenvironmental adaptations create a dynamic environment that fosters tumor growth and invasion.

## 3. Inflammatory Effects on Pituitary Adenoma Microenvironment and the HPA

Chronic inflammation induced by environmental pollutants, including airborne particulate matter, may contribute to dysregulated cellular proliferation in pituitary tissues. Exposure to airborne toxins and pro-inflammatory mediators can trigger oxidative stress, DNA damage, and epigenetic modifications, leading to genetic instability and aberrant cell cycle control [20]. This inflammatory microenvironment may interact with inherent genetic predispositions, amplifying oncogenic signaling pathways. Well-known genetic mutations linked to adenoma development, such as MEN1, AIP, and GNAS (see list of abbreviations), may be influenced by chronic inflammatory stimuli, further driving uncontrolled pituitary cell proliferation [21]. Additionally, the dysregulation of cell cycle proteins such as cyclin D1, along with an increased expression of anti-apoptotic proteins, further contribute to unchecked cellular growth and tumorigenesis [22]. These mechanisms, compounded by inflammatory and hypoxic conditions, may foster neoplastic transformation and tumor progression.

Immune cell infiltration and cytokine release within the TME drive chronic inflammation, promoting tumor growth. Elevated expression of endothelin-1 (ET-1), a vasoconstrictor peptide with pro-angiogenic and pro-tumorigenic effects, has been observed in various tumors. A study by Weindl et al. demonstrated that ET-1 acts as a significant growth factor in meningiomas, promoting cellular proliferation through the endothelin A receptor. While specific studies on ET-1 expression in invasive pituitary macroadenomas are limited, the role of ET-1 in tumorigenesis suggests potential implications for pituitary adenomas [23]. An additional factor to consider is the role of inducible nitric oxide synthase (iNOS), which is upregulated in pituitary adenomas under inflammatory conditions, enhancing vascular permeability and promoting tumor progression through hypoxia and oxidative stress [24]. Inflammatory cytokines, including IL-1β, IL-6, and TNF-α, amplify these effects by upregulating ET-1 and iNOS and activating pathways such as JAK/STAT and NF-κB, driving cellular proliferation, angiogenesis, and resistance to apoptosis [25].

The HPA axis is particularly vulnerable to inflammation due to its vascularization and regulatory functions. Cytokines like IL-1β and TNF-α can cross the blood–brain barrier (BBB) in regions with incomplete barriers, such as the median eminence, or act via the vascular endothelium to influence hypothalamic neurons. These cytokines activate toll-like receptors (TLRs) and downstream NF-κB signaling, increasing corticotropin-releasing hormone (CRH) production. Elevated CRH overstimulates the HPA axis, resulting in hypercortisolism and systemic hormonal imbalances.

An overexpression of inflammatory mediators such as IL-6 and TNF-α also disrupts the release of hypothalamic and pituitary trophic hormones and activates the JAK/STAT and MAPK pathways in anterior pituitary cells, leading to a dysregulated secretion of ACTH, GH, and prolactin [26,27]. Chronic inflammation and oxidative stress further impair pituitary responsiveness to hypothalamic signals, with increased iNOS expression causing nitric oxide-mediated damage and reducing hormonal output.

Chronic brain inflammation, white matter abnormalities, and microglia activation may result from exposure to air pollution, increasing the risk of autism spectrum disorders, neurodegenerative diseases, stroke, and multiple sclerosis [1]. Several theories have been proposed regarding the direct impact of air pollutants on the CNS. One theory suggests that respiratory exposure to air pollutants induces oxidative stress, increasing the permeability of the epithelial wall and triggering the release of pro-inflammatory cytokines, promoting auto-reactive T cell activation and enhancing their migration into the CNS via the blood–brain barrier (BBB) [28,29,30,31].

**Figure 1:**
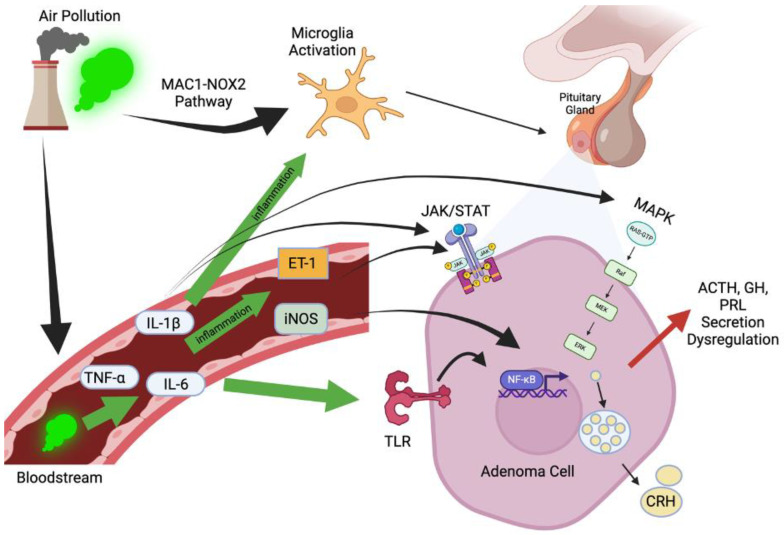
Air pollution enters the bloodstream and triggers inflammation, upregulating inflammatory cytokines, including TNF-α, IL-6, and IL-1β. The cytokines activate microglia and particles directly stimulate microglia, which in turn directly propagates CNS and HPA axis inflammation. The cytokines also upregulate iNOS and ET-1, as well as stimulating numerous inflammatory pathways, including JAK/STAT, MAPK, TLR, and NF-κB, which cause hormonal dysregulation affecting trophic hormones and directly affecting anterior pituitary hormone production.

According to the neuroinflammation hypothesis, increased cytokines and reactive oxygen species in the brain mediate the harmful effects of urban air pollution on the CNS. Microglia, a prominent source of cytokines and reactive oxygen species in the brain, contribute to progressive neuronal damage in various neurodegenerative diseases and are activated by inhaled urban air pollutants through both direct and indirect pathways. A key mechanism by which microglia respond to various forms of air pollution is the MAC1-NOX2 pathway, suggesting a potential common pathway [2]. Air pollution can contribute to toxic microglial activation by triggering the cycle of reactive microgliosis through three mechanisms: (1) components of air pollution may directly activate microglia; (2) cytokines from the peripheral systemic inflammatory response may activate microglia; (3) particles or adsorbed compounds derived from the periphery may directly damage neurons and cause reactive microgliosis [5]. A summary figure, Figure 1, is shown below.

## 4. Air Pollution and Pituitary Tumors

**Figure 2:**
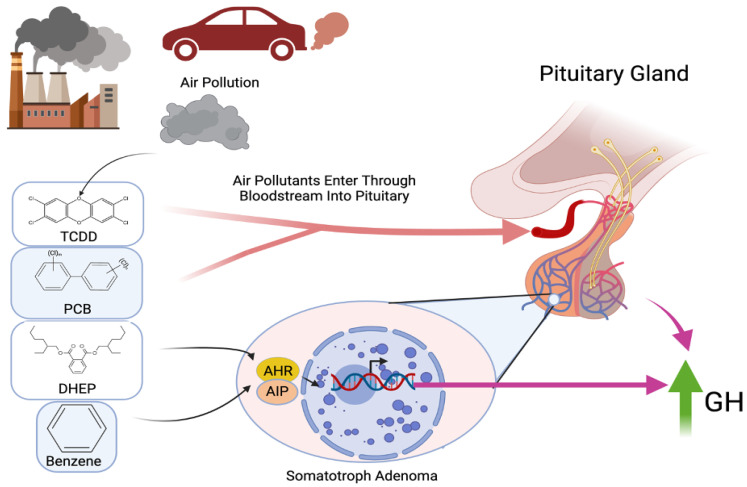
Schematic illustration depicting the proposed mechanistic link between air pollution exposure and growth hormone dysregulation in pituitary tumorigenesis, mediated through endocrine disruption at the level of the pituitary gland. Environmental pollutants, including TCDD, PCB, DHEP, and benzene, activate AHR and AIP in somatotroph cells, leading to increased GH secretion, which may contribute to tumor progression pathophysiology.

The first description of environmental pollutants’ detrimental effects on the pituitary dates back to the late 1950s, when Iannacone and Cicchella (1958) identified histological changes associated with benzene intoxication in rats [32]. More recently, a modest increase in the incidence of pituitary tumors was reported in the population of Seveso (Italy), exposed thirty years earlier to intermediate-to-high concentrations of 2,3,7,8-tetrachlorodibenzo-p-dioxin, a toxic and carcinogenic agent released in an industrial accident in 1976 and known to activate the aryl hydrocarbon receptor, whose interaction with aryl hydrocarbon receptor-interacting protein has been associated with the development of familial pituitary tumors [33,34]. In a study by Cannavò et al. (2010), a significantly higher rate of acromegaly was observed in a highly industrialized area near Messina (Italy) compared to more distant regions and the general population, with no link to genetic or familial predisposition. The involvement of environmental pollutants in the pathogenesis of somatotropinomas was then hypothesized [35]. Tapella et al. (2017) demonstrated in vitro that the pollutants benzene (BZ) and di(2-ethylhexyl) phthalate (DEHP) can interfere with normal rat pituitary cell proliferation and promote changes in gene expression at the aryl hydrocarbon receptor (AHR) and in aryl hydrocarbon receptor-interacting protein (AIP) levels, providing a link between epidemiological and genomic findings in pituitary tumors [36]. A study by Fortunati et al. aimed to identify the effects of pollutants, including benzene (BZ), bis(2-ethylhexyl) phthalate (DEHP), and polychlorinated biphenyls (PCBs), on the function of a GH-producing pituitary adenoma cell line (GH3) by analyzing gene and protein expression. Notably, they found that all of the studied pollutants increased GH production and secretion [33]. This is particularly significant as it represents the first demonstration that exposure to BZ, DEHP, and PCBs can enhance GH production in GH-producing adenoma cells. The proposed mechanistic link between air pollution exposure and pituitary dysfunction is illustrated in Figure 2, which highlights how environmental pollutants such as TCDD, PCB, DEHP, and benzene may activate AHR in somatotroph cells, leading to increased GH secretion and potentially influencing the pathogenesis and aggressiveness of somatotropinomas (Figure 2).

Consequently, the authors suggest that air pollution may alter the behavior of functioning pituitary adenomas, potentially making them more aggressive. Fortunati et al. also demonstrated that these pollutants can modulate the expression of somatostatin receptor 2 (SSTR2), along with the transcription factors (e.g., ZAC1 and FOXA1) involved in somatostatin analog (SSA) and estrogen hormone signaling [33]. The observed increase in FOXA1 expression could contribute to increased GH secretion by these cells, and together with the effect on ZAC1, it suggests that air pollutants may directly affect transcription regulators, thereby modulating cell function at multiple levels.

In addition to oxidative stress and AHR-mediated signaling, several classical molecular mechanisms may underlie pollutant-induced tumorigenesis. These include direct DNA damage or indirect genotoxicity via reactive metabolite formation, epigenetic modifications such as altered DNA methylation, and a dysregulation of pathways controlling cell proliferation and apoptosis. The disruption of nuclear hormone receptors, including estrogen and thyroid receptors, further contributes to endocrine imbalance and altered cellular homeostasis. While direct evidence linking these mechanisms to pituitary adenoma development is limited, their involvement in other endocrine and CNS tumors supports their plausibility and highlights key targets for future mechanistic investigation.

## 5. Public Health Implications, Challenges, and Future Directions

Although epidemiological studies are limited, recent reports suggest a link between environmental factors and pituitary adenomas, with an increased prevalence observed in highly industrialized areas and potentially following toxic spillage [34,36]. Tapella et al.’s study is the first to demonstrate that pollutants can induce proliferation in normal pituitary cells, bridging the gap between epidemiological and genomic findings in pituitary tumors. Their findings suggest that benzene and 2-ethyl-phthalate activate the AhR/AIP pathway, leading to enhanced proliferation in normal rat pituitary cells [35]. Experimental data support the findings of Tapella et al., highlighting an association between endocrine pollutants and their effects on cell lines derived from rat pituitary neoplasms, particularly estrogen-sensitive somatotropes (GH3) and mammosomatotropes (MtT/E-2) [35]. Other pollutants, including bisphenol A, genistein, o,p′-DDT, cadmium, and endosulfan, have also been shown to promote proliferation in these cell lines [37,38,39,40,41].

Despite the relatively small number of studies directly evaluating the effects of air pollution on pituitary adenoma pathogenesis, it is well known that air pollution poses significant health risks in many different areas, and it is clear from the reports detailed in this review that the pituitary gland and HPA axis are not spared. Air pollution is associated with a wide range of noncommunicable diseases affecting various organ systems, including lung cancer, chronic obstructive pulmonary disease (COPD), cardiovascular diseases, stroke, and other malignancies, such as bladder cancer and childhood leukemia [42]. Particulate matter (PM_2.5_ and PM_10_) and gaseous pollutants like ozone, nitrogen oxides, and sulfur dioxide can penetrate the respiratory system, leading to acute and chronic respiratory conditions, including asthma and bronchitis [43]. Neurological impacts are also significant, with air pollution linked to cognitive decline, dementia, and neuropsychiatric disorders through mechanisms involving neuroinflammation and disruption of the blood–brain barrier [44]. The systemic effects of air pollution are mediated through complex pathways involving oxidative stress, inflammation, and neuroendocrine regulation, which can exacerbate pre-existing conditions and lead to multi-organ damage [45,46].

It is currently estimated that nearly one half of the world’s entire population is potentially exposed to increasing levels of air pollution, and current research on the effects of climate change on air pollution predicts that air quality will further worsen as global temperatures rise [47,48]. In high-pollution areas, such as Nanjing, China, a study found that a 10 μg/m^3^ increase in particulate matter (PM) concentration was significantly associated with increased hospital outpatient visits for endocrine, digestive, urological, and dermatological diseases. Specifically, the increase in PM concentration was associated with a 0.59% increase in endocrine-related visits, highlighting the systemic impact of air pollution on endocrine health [49]. In contrast, studies in regions with lower pollution levels, such as the United States and Canada, have also documented adverse health effects at levels below current air quality standards. The Health Effects Institute (HEI) found positive associations between long-term exposure to fine particulate matter (PM_2.5_) and increased mortality, even at concentrations below the U.S. National Ambient Air Quality Standards. This suggests that even low levels of air pollution can have significant health impacts, including on cardiovascular and respiratory morbidity [50].

Following inhalation, volatile organic compounds such as benzene and particulate-bound toxins like PCBs and TCDD are absorbed through the respiratory epithelium and enter systemic circulation [3,5]. Lipophilic pollutants readily cross the blood–brain barrier either directly or through pollutant-induced vascular inflammation [3,5]. Toxicokinetic studies have demonstrated that compounds like benzene undergo hepatic metabolism via cytochrome P450 enzymes [51], generating reactive intermediates capable of inducing oxidative stress and DNA damage [51,52,53]. Similarly, DEHP and PCBs disrupt endocrine homeostasis through nuclear receptor interactions [54,55]. Dose–response relationships for these pollutants suggest that the environmental exposure levels commonly encountered in urban regions may approach biologically active thresholds [53,54]. Epidemiologic data link chronic exposure to benzene concentrations of 1–5 ppb with increased risks of endocrine dysfunction [54], while PM_2.5_-associated pollutants correlate with neuroinflammatory and endocrine effects even at low concentrations [55]. Mechanistic studies further demonstrate pollutant activation of AHR signaling in pituitary cell lines, promoting hormone secretion and proliferative signaling [33,35].

While occupational exposure limits such as Threshold Limit Values (TLVs) and Permissible Exposure Limits (PELs) have been established for select pollutants like benzene and DEHP, direct studies on pituitary-specific toxicological thresholds are lacking. Biomarkers of exposure, including trans, trans-muconic acid for benzene and MEHP for DEHP, provide evidence of systemic uptake, but their relevance to pituitary accumulation remains uncertain. Additionally, the potential for synergistic effects among co-exposures is biologically plausible yet uncharacterized in the context of pituitary tumorigenesis. These gaps underscore the need for future toxicological studies that specifically evaluate pituitary susceptibility to complex environmental exposures.

Simply put, the potential public health implications are astounding, and as further research better elucidates the relationship between air pollutants and functioning pituitary tumors, we suspect the impact may be significant. We again emphasize that further research is necessary to parse out the ways in which air pollutants facilitate neuroinflammation and pituitary tumorigenesis, and while this may be made more difficult by the immense number of individual pollutants present in our air today, it is evident that their influence cannot be ignored.

Moreover, it is important to note that much can be done to mitigate the harmful effects of air pollution on the pituitary gland, and other body systems for that matter, despite our ongoing efforts to better understand the underlying relationships. We suggest that regions with elevated pollution levels adopt stricter air quality regulations, including setting lower permissible limits for key pollutants, enhancing monitoring systems, and enforcing penalties for non-compliance. Investment should also be directed toward expanding clean energy initiatives, such as solar, wind, and hydroelectric power, and incentivizing industries and households to transition away from fossil fuels. Improved urban planning is another critical area, including increasing green spaces, implementing emission control zones, and designing cities to reduce vehicle traffic and promote public transportation, cycling, and walking. On an individual level, residents in high-pollution areas can take proactive measures to protect their health. These include wearing high-efficiency masks (such as N95 or equivalent respirators) when outdoors, especially during peak pollution periods, using indoor air purifiers, sealing homes to prevent outdoor air infiltration, and staying informed about daily air quality reports to adjust activities accordingly. Public education campaigns can further empower communities by raising awareness of pollution risks and available protective strategies.

Future studies should build upon the experimental findings detailed herein with larger sample sizes to help validate and strengthen the associations. Multigenerational research in exposed areas can be performed to better understand the long-term effects of endocrine pollutants on pituitary tumor development. Finally, comparisons of the rate of growth, tumor size, and pathological hormone levels between patients in pollutant-poor and pollutant-rich areas may further shed light on the magnitude of air pollution’s effect.

### Limitations

Given the emerging nature of this research area, several limitations exist within the discussion and studies cited in this review. We critically discuss the limitations of existing in vitro, in vivo, and epidemiological evidence, the current lack of definitive causal data, and key hypotheses that require future investigation. Many of the studies reviewed were conducted in animal models, which, while valuable for understanding biological mechanisms, may not fully reflect human physiology or disease processes. As a result, the applicability of these findings to human populations remains uncertain. Another limitation is the strength of the evidence supporting a causal link between air pollution and pituitary adenoma pathogenesis. While several studies suggest a potential association, the evidence remains inconclusive and does not yet establish a definitive causal relationship. Further research, particularly longitudinal human studies, is needed to strengthen these findings and clarify the potential mechanisms involved. The wide range of pollutants and their complex interactions make it challenging to fully understand their collective impact on tumorigenesis, underscoring the need for future research to clarify these mechanisms. Additionally, the differing pollution levels across geographic regions create challenges in making direct comparisons, highlighting the importance of conducting localized studies.

## 6. Conclusions

Emerging evidence suggests a compelling link between air pollution and the development of pituitary tumors. While traditionally viewed through the lens of respiratory and cardiovascular harm, air pollution’s potential neuroendocrine effects are becoming increasingly apparent. Chronic exposure to particulate matter and toxic pollutants may contribute to endocrine disruption, oxidative stress, inflammation, and even direct mutagenesis within the pituitary gland. Although current studies indicate a plausible biological pathway and epidemiological associations, research remains limited by small cohort sizes, variable exposure assessments, and a lack of mechanistic clarity. Future investigations must prioritize longitudinal, population-based studies and explore molecular mechanisms in greater detail to definitively establish causality. Understanding these connections not only deepens our knowledge of pituitary tumor pathogenesis but also highlights the urgent need for environmental health interventions to mitigate broader public health risks. Given the rising burden of both air pollution and pituitary disorders globally, this intersection represents a critical frontier in preventive medicine and neuroendocrine research.

## Data Availability

No new data were created or analyzed in this study.

## Abbreviations

The following abbreviations are used in this manuscript:

ACTH	Adrenocorticotropic hormone
AHR	Aryl hydrocarbon receptor
AIP	Aryl hydrocarbon receptor-interacting protein
ADH	Antidiuretic hormone
BBB	Blood–brain barrier
BZ	Benzene
CNS	Central nervous system
CRH	Corticotropin-releasing hormone
DEHP	Di(2-ethylhexyl) phthalate
ET-1	Endothelin-1
FSH	Follicle-stimulating hormone
GH	Growth hormone
GH3	GH-producing pituitary adenoma cell line
GNAS	Guanine Nucleotide-Binding Protein, Alpha Subunit
HIF-1α	Hypoxia-inducible factor 1-alpha
HPA	Hypothalamic–pituitary–adrenal
IL	Interleukin (e.g., IL-1β, IL-6)
iNOS	Inducible nitric oxide synthase
JAK/STAT	Janus Kinase/Signal Transducer and Activator of Transcription Pathway
LH	Luteinizing hormone
MAC1-NOX2	Macrophage Antigen 1–NADPH Oxidase 2 Pathway
MAPK	Mitogen-Activated Protein Kinase
MEN1	Multiple Endocrine Neoplasia Type 1
MMP	Matrix metalloproteinase
NF-κB	Nuclear Factor Kappa-light-chain-enhancer of Activated B Cells
PCBs	Polychlorinated biphenyls
ROS	Reactive oxygen species
SSA	Somatostatin analog
SSTR2	Somatostatin receptor 2
TME	Tumor microenvironment
TNF-α	Tumor Necrosis Factor-alpha
MtT/E-2	Mammosomatotrope Cell Line
